# Plasma immunoprofiling of patients with high-risk diffuse large B-cell lymphoma: a Nordic Lymphoma Group study

**DOI:** 10.1038/bcj.2016.113

**Published:** 2016-11-18

**Authors:** F Pauly, K Fjordén, S Leppä, H Holte, M Björkholm, Ø Fluge, L Møller Pedersen, M Eriksson, A Isinger-Ekstrand, C A K Borrebaeck, M Jerkeman, C Wingren

**Affiliations:** 1 Department of Immunotechnology, Lund University, Lund, Sweden; 2 CREATE Health, Lund University, Lund, Sweden; 3 Department of Oncology, Lund University, Skåne University Hospital, Lund, Sweden; 4 Department of Oncology, Helsinki University Hospital, Helsinki, Finland; 5 Department of Oncology, Oslo University Hospital, Oslo, Norway; 6 Department of Medicine, Karolinska University Hospital, Stockholm, Sweden; 7 Department of Oncology, Haukeland University Hospital, Bergen, Norway; 8 Department of Hematology, Roskilde Hospital, Roskilde, Denmark

Diffuse large B-cell lymphoma (DLBCL) is the most common type of aggressive lymphoma. The disease is heterogeneous with respect to the clinical course and molecular findings, making the management of these patients challenging. Deepened understanding of DLBCL pathogenesis has opened novel possibilities for early patient prognostication, but a further improved stratification will be essential to allow for individualized, risk-adapted therapy. So far, few studies have addressed the plasma proteome in DLBCL. In a recent pilot study we showed that plasma immunoprofiling, using recombinant antibody microarrays, could be used to decipher DLBCL heterogeneity on the protein level, indicating potential novel patient subgroups that might be linked to survival.^1^ Here, we have expanded these immunoprofiling efforts by examining the immunoprofiles of DLBCL patients, using longitudinal plasma samples collected at the time of diagnosis and during the treatment. Our aim was to search for prognostic and potentially predictive protein profiles in plasma samples from DLBCL patients.

The study population consisted of 126 patients with high-risk *de novo* DLBCL or follicular lymphoma grade III, included in a prospective phase II clinical trial of the Nordic Lymphoma Group during 2004–2008.^2^ Treatment consisted of six cycles of R-CHOEP-14 followed by systemic central nervous system prophylaxis with one cycle high-dose cytarabine and one cycle high-dose methotrexate. In the present study, patients were selected based on available plasma samples taken at diagnosis as a baseline sample (BL, *n*=116), after cycle 3 (Cy3, *n*=61) and/or after the final cycle 8 (Cy8, *n*=58). Age- and gender-matched healthy controls (*n*=40) were included (Supplementary Table 1).

In recent years, several proteomic methods have been developed, revealing novel insights into the complex proteome. Recombinant antibody microarray is a high-throughput proteomic technique with the ability to detect multiplexed panels of both high- and low-abundant proteins in different biofluids, providing protein expression profiles that reflect the composition of the proteome.^3^ A summary of the method and data analysis used in the present study is given in Supplementary Method, Supplementary Table 2 and Supplementary Figure 1. So far, the use of recombinant antibody microarrays has defined disease-specific signatures for several solid tumors and inflammatory conditions, by targeting mainly immunoregulatory proteins thought to reflect alterations in the host immune response.^4, 5^ In addition, a recent pilot study using plasma samples from DLBCL patients revealed a protein immunoprofile of 23 plasma proteins that could be used to differentiate the patients into two subgroups with significantly different overall survival (OS).^1^ Here, our approach was to expand the immunoprofiling of DLBCL patients, but in addition also target plasma proteins that are connected to proliferation and survival of the tumor cells.

The results showed that plasma protein profiles could distinguish newly diagnosed DLBCL patients from healthy controls. The number of significantly deregulated proteins in baseline samples taken at diagnosis vs samples from healthy controls was 58 (Supplementary Figure 2). The top 15 most significantly deregulated proteins were mainly upregulated in the plasma samples from patients, including interleukin (IL)-13, MAPK2 and tyrosine-protein kinase BTK (BTK). Although it can be expected that several immunoregulatory proteins have a different expression in these two groups, it is interesting to look into some of the differently expressed proteins more closely, such as the B-cell-stimulating T-helper 2 cytokine IL-13, which was upregulated in DLBCL patients compared with controls. High levels of IL-13 were also reported in a recent study on DLBCL patients, compared with healthy controls.^6^ Moreover, BTK was upregulated in the plasma from DLBCL patients. Due to the prominent role in B-cell receptor signaling, and the promising clinical results obtained with BTK inhibitors,^7^ further exploration of the clinical relevance of BTK levels in the plasma of DLBCL patients is warranted.

The protein profiles indicated massive changes in the plasma proteome protein during treatment. In particular, large changes in the plasma proteome were observed upon start of treatment; the number of significantly deregulated proteins being 48 at BL vs Cy3, compared with only 1 when comparing Cy3 with Cy8, and 0 at BL vs Cy8 (Supplementary Figure 3). In the case of BL vs Cy3, the top 15 deregulated proteins were all upregulated at Cy3, and included both T-helper 1 and 2 cytokines, and the monocyte chemotactic protein-1 (MCP-1). MCP-1 has been reported to be overexpressed on mRNA and protein levels in aggressive lymphomas.^8^ Considering the given treatment with chemotherapy, corticosteroids, rituximab and granulocyte colony-stimulating factor, it is difficult to draw any firm conclusions from the changes in the plasma proteome seen during treatment. However, the dynamics are interesting and encourage future studies in the quest of clinically useful information from protein profiles in the plasma.

Further, we investigated whether protein profiles at the time of diagnosis could classify the patients according to selected clinical variables. The results indicated that biologically relevant and differently expressed proteins were observed when comparing patients with different aaIPI or different failure-free survival (FFS) (Supplementary Figure 4). For example, cyclin-dependent kinase 2 (CDK-2) and BTK were observed to be upregulated in patients with higher aaIPI and in patients with shorter FFS. CDK-2 is a crucial component of cell cycle control, reported to be elevated in several B-cell malignancies.^9^ CDK inhibitors represent a promising class of drugs under development for treatment of a range of tumors, including DLBCL.^10^

In order to correlate the total change in expression of each protein over the three time points (BL, Cy3 and Cy8) to selected clinical variables, a response feature analysis was performed. When comparing the patients who developed progression during treatment with those who did not, six significantly deregulated proteins were identified, among them IL-6 (Supplementary Figure 5). High levels of IL-6 in plasma have previously been correlated to unfavorable prognosis for DLBCL patients.^11^ Our findings indicate that changes in the plasma proteome might be a helpful tool for early evaluation of treatment response, allowing rapid changes in the treatment of patients not responding to first-line treatment.

On the basis of plasma samples taken at diagnosis, and the protein signature of 23 plasma proteins with prognostic impact found in a previous pilot study from our group,^1^ patients in the present study could be divided into two distinct subgroups, denoted DLBCLa and DLBCLb, by unsupervised hierarchical clustering. The proteins in the profile included immunoregulatory proteins such as T-helper cytokines, chemotactic proteins and complement factors. Survival analysis of the generated subgroups showed a tendency toward different survival in the two groups (Figure 1), supporting the findings of the pilot study regarding the prognostic impact of the protein signature and suggesting that plasma protein profiles of newly diagnosed DLBCL patients may contain valuable prognostic information. The 23 proteins included in the present protein profile were mainly upregulated in the subgroup of patients showing a trend toward worse prognosis and included MCP-1, IL-6, IL-10 and tumor necrosis factor-α (TNF-α) (Figure 1). In addition, IL-10 and TNF-α were found to be significantly upregulated in patients with short OS, defined as OS<12 months compared with OS>12, 24, 36, 48 or 60 months. To investigate the prognostic power of these two biomarkers, they were compared with aaIPI in a Cox survival model. Adding IL-10 or TNF-α to the aaIPI improved the model, although adding both simultaneously did so to a lesser extent, indicating that the two proteins provided collinear information (Table 1). IL-10 is an immunosuppressive, but also B-cell stimulating, cytokine.^12^ TNF-α is an immunostimulatory cytokine with also tumor-promoting effects.^13^ In accordance with our results, overexpression of both IL-10 and TNF-α has previously been correlated to poor prognosis of DLBCL patients.^14, 15^ Moreover, when combining the information regarding DLBCL subgroup with the aaIPI in a Cox survival model, the model was improved for both OS and FFS compared with using the aaIPI alone (Table 1).

Taken together, in this study we have expanded our immunoprofiling efforts of DLBCL by examining the immunoprofiles of DLBCL patients before and during treatment. The results revealed DLBCL-associated plasma protein profiles, with the potential to reflect both alterations in the host immune response and the molecular pathogenesis of the tumor cells. New candidate markers for prediction of treatment response and prognosis were identified, and provide a basis for future investigations. As this study was mainly aimed for discovery, our findings must be validated in independent patient cohorts, to further examine the use of plasma protein profiling as a novel possibility for prognostication and prediction of treatment response in DLBCL. Moreover, ongoing and future efforts to select further antibodies with high specificity and stable on-chip function will allow us to target a wider range of proteins, and enable an even more highly resolved view of the complex plasma proteome in DLBCL.

## Figures and Tables

**Figure 1:**
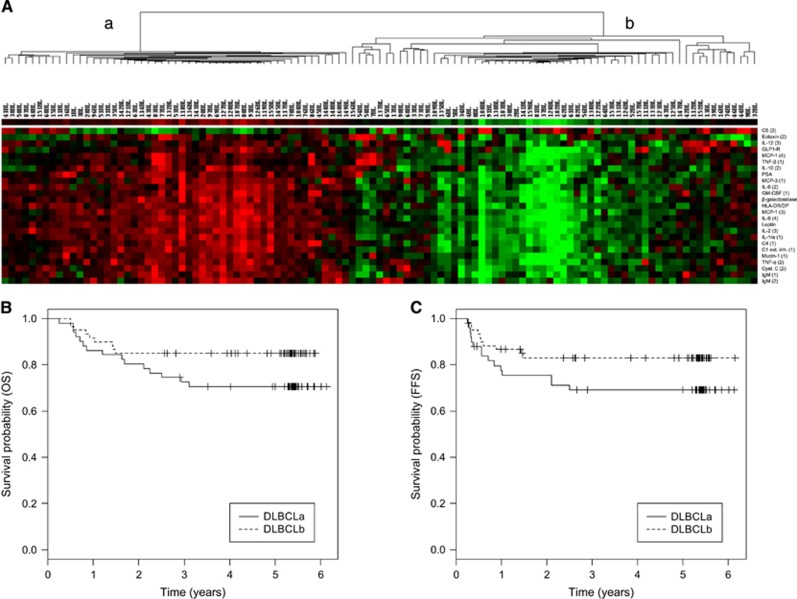
Stratification of DLBCL patients according to subgroup. (**A**) Subdivision of DLBCL patients into subgroups DLBCLa and DLBCLb based on a 23 biomarker signature identified in our previous study.^1^ (**B**) Kaplan–Meier plot demonstrating OS of DLBCLa and DLBCLb, log-rank *P*=0.07 for OS. (**C**) Kaplan–Meier plot demonstrating FFS of DLBCLa and DLBCLb, log-rank *P*=0.05 for FFS.

**Table 1:** Results of the Cox regression analysis

*Factor*	*Variables*	*HR*	*95% CI*	P*-value*
OS	aaIPI	2.2	1.0–4.7	0.05
	aaIPI	2.0	0.9–4.4	0.08
	TNF-a	1.5	0.2–9.8	0.70
	IL-10	3.0	0.6–15.5	0.20
	aaIPI	2.1	0.9–4.5	0.07
	TNF-a	3.4	0.9–13.1	0.08
	aaIPI	2.0	0.9–4.5	0.07
	IL-10	3.7	1.1–12.3	0.03
	aaIPI	2.2	1.0–4.8	0.05
	DLBCLa/b	1.9	0.9–3.9	0.11
FFS	aaIPI	2.0	1.0–4.4	0.06
	aaIPI	2.0	0.9–4.1	0.10
	TNF-a	1.5	0.2–9.7	0.67
	IL-10	2.8	0.6–14.6	0.21
	aaIPI	2.0	0.9–4.2	0.08
	TNF-a	3.3	0.9–12.7	0.08
	aaIPI	1.9	0.9–4.2	0.09
	IL-10	3.6	1.1–11.7	0.03
	aaIPI	2.1	1.0–4.6	0.05
	DLBCLa/b	2.0	1.0–4.3	0.06

Abbreviations: CI, confidence interval; DLBCL, diffuse large B-cell lymphoma; FFS, failure-free survival; HR, hazard ratio; IL, interleukin; OS, overall survival; TNF-α, tumor necrosis factor-α.

## Supplementary Material

Supplementary Method

## References

[bib1] Pauly F, Smedby KE, Jerkeman M, Hjalgrim H, Ohlsson M, Rosenquist R et al. Identification of B-cell lymphoma subsets by plasma protein profiling using recombinant antibody microarrays. Leuk Res 2014; 38: 682–690. 24754901 10.1016/j.leukres.2014.03.010

[bib2] Holte H, Leppa S, Bjorkholm M, Fluge O, Jyrkkio S, Delabie J et al. Dose-densified chemoimmunotherapy followed by systemic central nervous system prophylaxis for younger high-risk diffuse large B-cell/follicular grade 3 lymphoma patients: results of a phase II Nordic Lymphoma Group study. Ann Oncol 2013; 24: 1385–1392. 23247661 10.1093/annonc/mds621

[bib3] Borrebaeck CA, Wingren C. Antibody array generation and use. Methods Mol Biol 2014; 1131: 563–571. 24515491 10.1007/978-1-62703-992-5_36

[bib4] Wingren C, Sandstrom A, Segersvard R, Carlsson A, Andersson R, Lohr M et al. Identification of serum biomarker signatures associated with pancreatic cancer. Cancer Res 2012; 72: 2481–2490. 22589272 10.1158/0008-5472.CAN-11-2883

[bib5] Carlsson A, Wuttge DM, Ingvarsson J, Bengtsson AA, Sturfelt G, Borrebaeck CA et al. Serum protein profiling of systemic lupus erythematosus and systemic sclerosis using recombinant antibody microarrays. Mol Cell Proteomics 2011; 10: M110 005033. 10.1074/mcp.M110.005033 PMC3098590 21350050

[bib6] Charbonneau B, Maurer MJ, Ansell SM, Slager SL, Fredericksen ZS, Ziesmer SC et al. Pretreatment circulating serum cytokines associated with follicular and diffuse large B-cell lymphoma: a clinic-based case-control study. Cytokine 2012; 60: 882–889. 23010502 10.1016/j.cyto.2012.08.028 PMC3483382

[bib7] Young RM, Staudt LM. Targeting pathological B cell receptor signalling in lymphoid malignancies. Nat Rev Drug Discov 2013; 12: 229–243. 23449308 10.1038/nrd3937 PMC7595252

[bib8] Valkovic T, Duletic-Nacinovic A, Stifter S, Hasan M, Hadzisejdic I, Zombori D et al. Macrophage chemotactic protein-1 mRNA levels in non-Hodgkin lymphoma. Clin Exp Med 2010; 10: 229–235. 20232106 10.1007/s10238-010-0093-6

[bib9] Al-Assar O, Rees-Unwin KS, Menasce LP, Hough RE, Goepel JR, Hammond DW et al. Transformed diffuse large B-cell lymphomas with gains of the discontinuous 12q12-14 amplicon display concurrent deregulation of CDK2, CDK4 and GADD153 genes. Br J Haematol 2006; 133: 612–621. 16704435 10.1111/j.1365-2141.2006.06093.x

[bib10] Pitts TM, Davis SL, Eckhardt SG, Bradshaw-Pierce EL. Targeting nuclear kinases in cancer: development of cell cycle kinase inhibitors. Pharmacol Therap 2014; 142: 258–269. 24362082 10.1016/j.pharmthera.2013.12.010

[bib11] Giachelia M, Voso MT, Tisi MC, Martini M, Bozzoli V, Massini G et al. Interleukin-6 plasma levels are modulated by a polymorphism in the *NF-kappaB1* gene and are associated with outcome following rituximab-combined chemotherapy in diffuse large B-cell non-Hodgkin lymphoma. Leuk Lymphoma 2012; 53: 411–416. 21902578 10.3109/10428194.2011.621566

[bib12] Moore KW, de Waal Malefyt R, Coffman RL, O'Garra A. Interleukin-10 and the interleukin-10 receptor. Annu Rev Immunol 2001; 19: 683–765. 11244051 10.1146/annurev.immunol.19.1.683

[bib13] Wu Y, Zhou BP. TNF-alpha/NF-kappaB/Snail pathway in cancer cell migration and invasion. Br J Cancer 2010; 102: 639–644. 20087353 10.1038/sj.bjc.6605530 PMC2837572

[bib14] Gupta M, Han JJ, Stenson M, Maurer M, Wellik L, Hu G et al. Elevated serum IL-10 levels in diffuse large B-cell lymphoma: a mechanism of aberrant JAK2 activation. Blood 2012; 119: 2844–2853. 22323454 10.1182/blood-2011-10-388538 PMC3327462

[bib15] Lech-Maranda E, Bienvenu J, Broussais-Guillaumot F, Warzocha K, Michallet AS, Robak T et al. Plasma TNF-alpha and IL-10 level-based prognostic model predicts outcome of patients with diffuse large B-cell lymphoma in different risk groups defined by the International Prognostic Index. Arch Immunol Therap Exp 2010; 58: 131–141. 10.1007/s00005-010-0066-1 20191326

