# Supplementary material for: Plasma immunoprofiling of patients with high-risk diffuse large B-cell lymphoma: a Nordic Lymphoma Group study
Source: Blood Cancer J. 2016 Nov 18;6(11):e501–. doi: 10.1038/bcj.2016.113 (PMC5148057; doi:10.1038/bcj.2016.113)
Supplement: Supplementary Method [file bcj2016113x1.docx]

**Supplemental materials and methods**

**Antibodies**

Two hundred and eighty-three recombinant scFv antibody fragments directed against 97 known serum antigens and 29 short amino acid motifs denoted CIMS 1-29 (4 to 6 residues long) were selected from phage display libraries ([1](#_ENREF_1)) (Persson et al, submitted 2016) (Supplemental Table 1). Specificity and on-chip functionality of the antibodies has been ensured by a wide range of different approaches, involving the antibody design, antibody selection, and validation of the specificity with orthogonal methods (Supplemental Table 1).

All scFv antibodies were produced in *E*. *coli* cultures and purified from the periplasm using a MagneHis Protein Purification system (Promega, Madison, WI, USA) and a KingFisher96 robot (Thermo Fisher Scientific, Waltham, MA, USA). The elution buffer was exchanged for PBS using Zeba 96-well desalt spin plates (Pierce). The protein concentration was measured by absorbance at 280nm using NanoDrop (Thermo Scientific, Wilmington, DE, USA) and the purity of the scFv antibodies was evaluated using 10% SDS-PAGE (Invitrogen, Carlsbad, CA, USA).

**Antibody microarrays**

For production of the antibody microarrays, we used a setup recently optimized ([2-4](#_ENREF_2)) (Delfani et al, submitted 2016). The antibody microarrays were printed on black Maxisorp slides (NUNC, Roskilde, Denmark) using a non-contact printer (SciFlexarrayer S11, Scienion, Berlin, Germany). Thirteen identical subarrays consisting of 31x33 spots were printed onto each slide, and every antibody was printed in 3 replicates to assure adequate reproducibility (Supplemental Figure 1). Antibody spots measured 140 µm in diameter, and were spotted at a pitch-to-pitch distance of 200 µm. Ten slides, i.e. in total 130 subarrays, were printed every day for three days, and five slides, i.e. in total 65 subarrays, were printed on day four. All printing was performed overnight and the slides were used for array analysis the following day.

Each slide was mounted in a hybridization gasket (Schott, Jena, Germany) and blocked with PBS–MT (1% (w/v) milk, 1% (w/v) Tween-20 in PBS) for one hour. Labeled samples were diluted 1:10 in PBS-MT with 4mM EDTA. The slides were washed with PBS-T (0.05% (w/v) Tween-20 in PBS) four times before addition of sample, followed by incubation for two hours on an orbital rocking table. The slides were subsequently washed four times with PBS-T before addition of 1 µg/ml Alexa-647 Streptavidin in PBS-MT and incubated for one hour on the orbital rocking table. The slides were finally washed four times with PBS-T, immersed in dH2O and dried under a stream of N2 gas before being immediately scanned using a confocal microarray scanner (PerkinElmer Life and Analytical Sciences, Wellesley, MA, USA) at 10 µm resolution using 60% PMT gain and 90% laser power. Signal intensities were quantified using the ScanArray Express Software v4.0 (PerkinElmer Life and Analytical Sciences) using the fixed circle method. Further data analysis was performed on intensity values corrected for local background.

**Data pre-processing**

Data pre-processing was performed according to an approach recently optimized (Delfani et al, submitted 2016). Briefly, an average value of the three replicate spots spread out over the array was used unless any replicate CV exceeded 15% from the mean value, in which case the worst performing replicate was eliminated and the average value of the two remaining replicates was used instead. In this manner, 88% of the data values were calculated from all three replicate spots, and the remaining 12% from two replicates. The average replicate CV was 7%. Any antibodies from which signal intensities were found to be below limit of detection (LOD), defined as the average blank signal (PBS) plus two standard deviations, in >30% of samples were removed, resulting in the removal of one antibody, IL-3 (3).

For evaluation of normalization strategies, the data was visualized using principal component analysis (PCA) and hierarchical clustering (Qlucore, Lund, Sweden). PCA on log2 raw data showed, as was expected, some systematic differences between days of analysis, and minor systematic differences between arrays within in the same day of analysis. These differences were handled by normalization carried out in two steps. First, the differences between days of analysis were eliminated using a subtract by group mean strategy ([5](#_ENREF_5)) thus centering the data. In the next step, the array to array differences were removed by using a semi-global normalization approach reported earlier ([6](#_ENREF_6), [7](#_ENREF_7)) with the modification of using standard deviation (SD) instead of CV as a measure of variance.

**Data analysis**

Analysis of the samples was conducted using three different approaches, cases versus controls (BL vs. N, Cy3 vs. N, and Cy8 vs. N), within-case analysis (subgroups of BL/Cy3/Cy8 vs. subgroups of BL/Cy3/Cy8) and longitudinal analyses (BL vs. Cy3, BL vs. Cy8, and Cy3 vs. Cy8). When classifying cases from controls and performing within-cases analysis, Support Vector Machine (SVM) analysis was performed in R ([www.r-project.org](http://www.r-project.org)) using a linear kernel with the cost of constraints set to one (default value). The SVM was trained using a leave-one-out cross-validation procedure, and no filtration on the data was done before training, *i.e.* all antibodies used on the microarray were included in the analysis. Further, a receiver operating characteristics (ROC) curve, as constructed using the SVM decision values and the area under the curve (AUC), was calculated. AUC values were interpreted as 0.5-0.6 = poor; 0.6-0.7 = fair; 0.7-0.8 = intermediate; 0.8-0.9 = good; 0.9-1.0 = excellent. Significantly up- or down-regulated plasma proteins (*P*<0.05) were identified using the Wilcoxon test.

When performing the longitudinal analysis, paired *t*-tests were performed to identify significantly up- or down-regulated plasma proteins (*P*<0.05) between time points. In addition, in order to analyze the entire time course as one outcome and correlate this to clinical parameters, response feature analysis was performed using area under the curve (AUC) as response feature and *t*-test as method of analysis, identifying significantly up- or down-regulated plasma proteins (*P*<0.05) between patient groups. All *P* values were adjusted for multiple comparisons using the Benjamini-Hochberg method ([8](#_ENREF_8)).

Finally, in order to evaluate if results found in a previous study on B-cell lymphomas could be repeated, where two subgroups of DLBCL (denoted DLBCLa and DLBCLb) correlating to survival were identified ([9](#_ENREF_9)), an unsupervised hierarchical clustering method was applied. In brief, data from all the samples in the current study were mean centered before being hierarchically clustered according to the proteins which separated the two groups most efficiently in the previous study. The data was visualized as heat maps using Cluster and TreeView ([10](#_ENREF_10)). Survival analyses were performed on the resulting subgroups in this study. Kaplan-Meier plots were constructed and significance between groups was tested using the log-rank test, based on our recent discovery finding, a one-tailed log-rank test was used (9). Cox proportional hazards regression analysis was also performed, comparing the prognostic value of the aaIPI with the subgroups (denoted DLBCLa and DLBCLb), along with two analytes found to correlate significantly with survival in both the current and the previous study, IL-10 and TNF-α.

**REFERENCES**

1. Soderlind E, Strandberg L, Jirholt P, Kobayashi N, Alexeiva V, Aberg A-M, et al. Recombining germline-derived CDR sequences for creating diverse single-framework antibody libraries. Nat Biotech. 2000;18(8):852-6.

2. Ingvarsson J, Larsson A, Sjöholm AG, Truedsson L, Jansson B, Borrebaeck CAK, et al. Design of Recombinant Antibody Microarrays for Serum Protein Profiling:  Targeting of Complement Proteins. Journal of Proteome Research. 2007;6(9):3527-36.

3. Carlsson A, Persson O, Ingvarsson J, Widegren B, Salford L, Borrebaeck CA, et al. Plasma proteome profiling reveals biomarker patterns associated with prognosis and therapy selection in glioblastoma multiforme patients. Proteomics Clinical applications. 2010;4(6-7):591-602.

4. Wingren C, Ingvarsson J, Dexlin L, Szul D, Borrebaeck CAK. Design of recombinant antibody microarrays for complex proteome analysis: Choice of sample labeling-tag and solid support. PROTEOMICS. 2007;7(17):3055-65.

5. Wu YW, Wooldridge PJ. The impact of centering first-level predictors on individual and contextual effects in multilevel data analysis. Nursing research. 2005;54(3):212-6.

6. Ingvarsson J, Wingren C, Carlsson A, Ellmark P, Wahren B, Engström G, et al. Detection of pancreatic cancer using antibody microarray-based serum protein profiling. PROTEOMICS. 2008;8(11):2211-9.

7. Carlsson A, Wingren C, Ingvarsson J, Ellmark P, Baldertorp B, Fernö M, et al. Serum proteome profiling of metastatic breast cancer using recombinant antibody microarrays. European journal of cancer (Oxford, England : 1990). 2008;44(3):472-80.

8. Benjamini Y, Hochberg Y. Controlling the False Discovery Rate: A Practical and Powerful Approach to Multiple Testing. Journal of the Royal Statistical Society Series B (Methodological). 1995;57(1):289-300.

9. Pauly F, Smedby KE, Jerkeman M, Hjalgrim H, Ohlsson M, Rosenquist R, et al. Identification of B-cell lymphoma subsets by plasma protein profiling using recombinant antibody microarrays. Leukemia Research. 2014(0).

10. Eisen MB, Spellman PT, Brown PO, Botstein D. Cluster analysis and display of genome-wide expression patterns. Proceedings of the National Academy of Sciences. 1998;95(25):14863-8.
