# Supplementary material for: Plasma immunoprofiling of patients with high-risk diffuse large B-cell lymphoma: a Nordic Lymphoma Group study
Source: Blood Cancer J. 2016 Nov 18;6(11):e501–. doi: 10.1038/bcj.2016.113 (PMC5148057; doi:10.1038/bcj.2016.113)
Supplement: Supplementary Table 1 [file bcj2016113x2.docx]

| Parameter | DLBCL | | | | | | |  | | | Controls | | |  |  |
| --- | --- | --- | --- | --- | --- | --- | --- | --- | --- | --- | --- | --- | --- | --- | --- |
|  | Total | Baseline^a^ | | Cycle 3^b^ | | Cycle 8^c^ | | | |  | | |  | | |
| No. of patients | 126 | | 116 | | 61 | | 58 | |  | | | 40 | | |  |
| Gender (M:F) | 78:48 | | 74:42 | | 39:22 | | 34:24 | |  | | | 27:13 | | |  |
| Median age at diagnosis (range) | 53 (18-65) | | 53 (18-65) | | 53 (18-64) | | 53 (26-64) | |  | | | 63 (46-74) | | |  |
| 5 year overall survival | 65% | | 66% | | 72% | | 67% | |  | | | - | | |  |
| Ann Arbor stage (1:2:3:4) | 0:3:52:71 | | 0:2:49:65 | | 0:2:27:32 | | 0:1:25:32 | |  | | | - | | |  |
| Performance status (0:1:2:3:4) | 25:59:30:12:0 | | 22:56:27:11:0 | | 12:30:14:5:0 | | 12:23:18:5:0 | |  | | | - | | |  |
| IPI (1:2:3:4) | 0:92:34:0 | 0:85:31:0 | | 0:48:13:0 | | 0:40:18:0 | | | |  | | | - | | |

**Suppl Table 1**. **Demographic data of the patients included in the study.**

| Parameter | DLBCL | | | | | | |  | | | Controls | | |  |  |
| --- | --- | --- | --- | --- | --- | --- | --- | --- | --- | --- | --- | --- | --- | --- | --- |
|  | Total | Baseline^a^ | | Cycle 3^b^ | | Cycle 8^c^ | | | |  | | |  | | |
| No. of patients | 126 | | 116 | | 61 | | 58 | |  | | | 40 | | |  |
| Gender (M:F) | 78:48 | | 74:42 | | 39:22 | | 34:24 | |  | | | 27:13 | | |  |
| Median age at diagnosis (range) | 53 (18-65) | | 53 (18-65) | | 53 (18-64) | | 53 (26-64) | |  | | | 63 (46-74) | | |  |
| 5 year overall survival | 65% | | 66% | | 72% | | 67% | |  | | | - | | |  |
| Ann Arbor stage (1:2:3:4) | 0:3:52:71 | | 0:2:49:65 | | 0:2:27:32 | | 0:1:25:32 | |  | | | - | | |  |
| Performance status (0:1:2:3:4) | 25:59:30:12:0 | | 22:56:27:11:0 | | 12:30:14:5:0 | | 12:23:18:5:0 | |  | | | - | | |  |
| IPI (1:2:3:4) | 0:92:34:0 | 0:85:31:0 | | 0:48:13:0 | | 0:40:18:0 | | | |  | | | - | | |

a - Sample taken at time of diagnosis

b - Sample taken after 3 cycles of treatment

c - Sample taken after 8 cycles of treatment

| Parameter | DLBCL | | | | | | |  | | | Controls | | |  |  |
| --- | --- | --- | --- | --- | --- | --- | --- | --- | --- | --- | --- | --- | --- | --- | --- |
|  | Total | Baselin | | Cycle 3^b^ | | Cycle 8^c^ | | | |  | | |  | | |
| No. of patients | 126 | | 116 | | 61 | | 58 | |  | | | 40 | | |  |
| Gender (M:F) | 78:48 | | 74:42 | | 39:22 | | 34:24 | |  | | | 27:13 | | |  |
| Median age at diagnosis (range) | 53 (18-65) | | 53 (18-65) | | 53 (18-64) | | 53 (26-64) | |  | | | 63 (46-74) | | |  |
| 5 year overall survival | 65% | | 66% | | 72% | | 67% | |  | | | - | | |  |
| Ann Arbor stage (1:2:3:4) | 0:3:52:71 | | 0:2:49:65 | | 0:2:27:32 | | 0:1:25:32 | |  | | | - | | |  |
| Performance status (0:1:2:3:4) | 25:59:30:12:0 | | 22:56:27:11:0 | | 12:30:14:5:0 | | 12:23:18:5:0 | |  | | | - | | |  |
| IPI (1:2:3:4) | 0:92:34:0 | 0:85:31:0 | | 0:48:13:0 | | 0:40:18:0 | | | |  | | | - | | |
