# Supplementary material for: Plasma immunoprofiling of patients with high-risk diffuse large B-cell lymphoma: a Nordic Lymphoma Group study
Source: Blood Cancer J. 2016 Nov 18;6(11):e501–. doi: 10.1038/bcj.2016.113 (PMC5148057; doi:10.1038/bcj.2016.113)
Supplement: Supplementary Table 2 [file bcj2016113x3.docx]

**Suppl Table 2.** **Proteins targeted by the recombinant antibody microarray.** The antibodies were selected from designed phage display libraries[^1^](#_ENREF_1) (Persson et al, 2014 ms in prep). The specificity, affinity (normally in the nM range), and on-chip functionality of these phage display derived scFv antibodies was ensured by using i) stringent phage-display selection and screening protocols,[^2^](#_ENREF_2) ii) multiple clones (1-4) per target, and iii) a molecular design, adapted for microarray applications.[^3^](#_ENREF_3) In addition, the specificity of several of the antibodies have previously also been validated using well-characterized, standardized serum samples (with known levels of the targeted analytes), and/or orthogonal methods, such as mass spectrometry (affinity pull-down experiments), ELISA, MesoScaleDiscovery (MSD) assay, cytometric bead assay, and MS, as well as using spiking and blocking experiments.[^4-12^](#_ENREF_4) Notably, the reactivity of some antibodies might be lost since the label (biotin) used to label the sample to enable detection could block the affinity binding to the antibodies (epitope masking), but we have bypassed this problem, as in this study, by frequently including more than one antibody against the same protein, but directed against different epitopes.[^3^](#_ENREF_3)

| Protein | Full name | No. of antibody clones |
| --- | --- | --- |
| Angiomotin | Angiomotin | 2 |
| Apo-A1 | Apolipoprotein A1 | 3 |
| Apo-A4 | Apolipoprotein A4 | 3 |
| ATP-5B | ATP synthase subunit beta, mitochondrial | 2 |
| β-galactosidase | Beta-galactosidase | 1 |
| BTK | Tyrosine-protein kinase BTK | 4 |
| C1 inhibitor | Plasma protease C1 inhibitor | 4 |
| C1q* | Complement C1q | 1 |
| C1s | Complement C1s | 1 |
| C3* | Complement C3 | 6 |
| C4* | Complement C4 | 4 |
| C5* | Complement C5 | 3 |
| CD40 | CD40 protein | 4 |
| CD40L | CD40 ligand | 1 |
| CDK-2 | Cyclin-dependent kinase 2 | 2 |
| CHX10 | Visual system homeobox 2 | 3 |
| CIMS | Context independent motifs (4 to 6 amino acids long) | 29 |
| CT | Cholera toxin subunit B | 1 |
| Cyst. C | Cystatin C | 4 |
| Digoxin | Digoxin | 1 |
| DUSP9 | Dual specificity protein phosphatase 9 | 1 |
| EGFR | Epidermal growth factor receptor | 1 |
| Eotaxin | Eotaxin | 3 |
| Factor B* | Complement factor B | 4 |
| FASN | Fatty acid synthase | 4 |
| GAK | Cyclin G-associated kinase | 3 |
| GLP-1 | Glucagon-like peptide-1 | 1 |
| GLP-1R | Glucagon-like peptide 1 receptor | 1 |
| GM-CSF | Granulocyte-macrophage colony-stimulating factor | 6 |
| HADH2 | 3-hydroxyacyl-CoA dehydrogenase type-2 | 4 |
| Her2/ErbB-2 | Receptor tyrosine-protein kinase erbB-2 | 4 |
| HLA-DR/DP | HLA-DR/DP | 1 |
| ICAM-1 | Intercellular adhesion molecule 1 | 1 |
| IFN-γ | Interferon gamma | 3 |
| IgM | Immunoglobulin M | 5 |
| IL-10* | Interleukin-10 | 3 |
| IL-11 | Interleukin-11 | 3 |
| IL-12* | Interleukin-12 | 3 |
| IL-13* | Interleukin-13 | 3 |
| IL-16 | Interleukin-16 | 3 |
| IL-18 | Interleukin-18 | 3 |
| IL-1α* | Interleukin-1 alpha | 3 |
| IL-1β | Interleukin-1 beta | 3 |
| IL-1ra | Interleukin-1 receptor antagonist protein | 3 |
| IL-2 | Interleukin-2 | 2 |
| IL-3 | Interleukin-3 | 2 |
| IL-4* | Interleukin-4 | 4 |
| IL-5* | Interleukin-5 | 3 |
| IL-6* | Interleukin-6 | 8 |
| IL-7 | Interleukin-7 | 2 |
| IL-8* | Interleukin-8 | 3 |
| IL-9 | Interleukin-9 | 3 |
| Integrin α-10 | Integrin alpha-10 | 1 |
| Integrin α-11 | Integrin alpha-11 | 1 |
| JAK3 | Tyrosine-protein kinase JAK3 | 1 |
| KIA0882 | TBC1 domain family member 9 | 3 |
| Keratin19 | Keratin, type I cytoskeletal 19 | 3 |
| KSYK | Tyrosine-protein kinase SYK | 2 |
| LDL | Apolipoprotein B-100 | 2 |
| Leptin | Leptin | 1 |
| Lewis x | Lewis x | 2 |
| Lewis y | Lewis y | 1 |
| LUM | Lumican | 1 |
| MAPK1 | Mitogen-activated protein kinase 1 | 4 |
| MAPK2 | Mitogen-activated protein kinase 8 | 3 |
| MATK | Megakaryocyte-associated tyrosine-protein kinase | 3 |
| MCP-1* | C-C motif chemokine 2 | 9 |
| MCP-3 | C-C motif chemokine 7 | 3 |
| MCP-4 | C-C motif chemokine 13 | 2 |
| Mucin-1 | Mucin-1 | 6 |
| MYOM2 | Myomesin-2 | 2 |
| OSTP | Osteopontin | 3 |
| OSPBL3 | Oxysterol-binding protein-related protein 3 | 2 |
| P85A | Phosphatidylinositol 3-kinase regulatory subunit alpha | 3 |
| PKB gamma | RAC-gamma serine/threonine-protein kinase | 2 |
| Procathepsin W | Procathepsin W | 1 |
| Properdin* | Properdin | 1 |
| PSA | Prostate-specific antigen | 1 |
| PTK-6 | Protein-tyrosine kinase 6 | 1 |
| PTP-B1 | Tyrosine-protein phosphatase non-receptor type 1 | 3 |
| RANTES | C-C motif chemokine 5 | 3 |
| RPS6KA2 | Ribosomal protein S6 kinase alpha-2 | 3 |
| Sialyl Lewis x | Sialyl Lewis x | 1 |
| STAP2 | Signal-transducing adaptor protein 2 | 4 |
| STAT1 | Signal transducer and activator of transcription 1-alpha/beta | 2 |
| TENS4 | Tensin-4 | 1 |
| TGF-β1 | Transforming growth factor beta-1 | 2 |
| TM peptide | Transmembrane peptide | 1 |
| TNF-α | Tumor necrosis factor | 3 |
| TNF-β* | Lymphotoxin-alpha | 4 |
| TNFRSF14 | Tumor necrosis factor receptor superfamily member 14 | 2 |
| TNFRSF3 | Tumor necrosis factor receptor superfamily member 3 | 3 |
| UBC9 | SUMO-conjugating enzyme UBC9 | 3 |
| UBE2C | Ubiquitin-conjugating enzyme E2 C | 2 |
| UCHL5 | Ubiquitin carboxyl-terminal hydrolase isozyme L5 | 1 |
| UPF3B | Regulator of nonsense transcripts 3B | 2 |
| VEGF* | Vascular endothelial growth factor | 4 |

* Antibody specificity determined by protein arrays, MSD, ELISA, blocking/spiking experiments, and/or mass spectrometry.

1. Ewert S, Huber T, Honegger A, Pluckthun A. Biophysical properties of human antibody variable domains. *J Mol Biol*. 2003;325(3):531-553.

2. Soderlind E, Strandberg L, Jirholt P, et al. Recombining germline-derived CDR sequences for creating diverse single-framework antibody libraries. *Nat Biotech*. 2000;18(8):852-856.

3. Borrebaeck CK, Wingren C. Recombinant Antibodies for the Generation of Antibody Arrays. In: Korf U, ed. Protein Microarrays. Vol. 785: Humana Press; 2011:247-262.

4. Ingvarsson J, Larsson A, Sjöholm AG, et al. Design of Recombinant Antibody Microarrays for Serum Protein Profiling:  Targeting of Complement Proteins. *Journal of Proteome Research*. 2007;6(9):3527-3536.

5. Kristensson M, Olsson K, Carlson J, et al. Design of recombinant antibody microarrays for urinary proteomics. *PROTEOMICS – Clinical Applications*. 2012;6(5-6):291-296.

6. Wingren C, Ingvarsson J, Dexlin L, Szul D, Borrebaeck CAK. Design of recombinant antibody microarrays for complex proteome analysis: Choice of sample labeling-tag and solid support. *PROTEOMICS*. 2007;7(17):3055-3065.

7. Persson J, Bäckström M, Johansson H, Jirström K, Hansson GC, Ohlin M. Molecular Evolution of Specific Human Antibody against MUC1 Mucin Results in Improved Recognition of the Antigen on Tumor Cells. *Tumor Biology*. 2009;30(4):221-231.

8. Gustavsson E, Ek S, Steen J, et al. Surrogate antigens as targets for proteome-wide binder selection. *New Biotechnology*. 2011;28(4):302-311.

9. Carlsson A, Wuttge DM, Ingvarsson J, et al. Serum Protein Profiling of Systemic Lupus Erythematosus and Systemic Sclerosis Using Recombinant Antibody Microarrays. *Molecular & Cellular Proteomics*. 2011;10(5).

10. Dexlin-Mellby L, Sandström A, Centlow M, et al. Tissue proteome profiling of preeclamptic placenta using recombinant antibody microarrays. *PROTEOMICS – Clinical Applications*. 2010;4(10-11):794-807.

11. Ingvarsson J, Wingren C, Carlsson A, et al. Detection of pancreatic cancer using antibody microarray-based serum protein profiling. *PROTEOMICS*. 2008;8(11):2211-2219.

12. Pauly F, Dexlin-Mellby L, Ek S, et al. Protein Expression Profiling of Formalin-Fixed Paraffin-Embedded Tissue Using Recombinant Antibody Microarrays. *J Proteome Res*. 2013;12;5943-5953.
