# Supplementary material for: Plasma immunoprofiling of patients with high-risk diffuse large B-cell lymphoma: a Nordic Lymphoma Group study
Source: Blood Cancer J. 2016 Nov 18;6(11):e501–. doi: 10.1038/bcj.2016.113 (PMC5148057; doi:10.1038/bcj.2016.113)
Supplement: Supplementary Figure Legends [file bcj2016113x4.docx]

**Supplementary figure legends**

**Figure S1**. A representative scanned microarray image of a recombinant antibody microarray hybridized with plasma from a DLBCL patient. Thirteen identical subarrays denoted 1A-F and 2A-G were spotted. Subarray D2 is enlarged, showing the array layout with 33x31 spots. The arrays consist of three segments separated by printed rows of labeled BSA (row 1, 11, 21 and 31). Each antibody was printed in three replicate spots, one in each segment.

**Figure S2**. Classification of DLBCL patients from controls. (A) Classification of DLBCL patients at BL, Cy3 and Cy8 from healthy controlls (N) using an SVM based approach expressed in terms of ROC AUC values. (B-D) Top 15 differentially expressed proteins (adjusted P<0.05) for the comparison BL, Cy3 and Cy8 with N respectively. Fold changes (FC) are presented in heat maps; red – upregulated, green – down-regulated and black – equal levels.

**Figure S3**. Immunoprofiles of DLBCL patients during the course of treatment. Top 25 significantly deregulated proteins (P<0.05) as determined by paired t-tests between time points (A) BL and Cy3, (B) Cy3 and Cy8 and (C) BL and Cy8 (only 23 deregulated proteins). Fold changes (FC) are presented in heat maps; red – upregulated, green – down-regulated and black – equal levels.

**Figure S4**. Correlation of immunoprofiles of DLBCL patients to clinical parameters at time of diagnosis. (A) Correlation of clinical parameters to immunoprofiles as determined by an SVM based analysis and expressed in terms of ROC AUC values. Lists of significantly deregulated proteins are presented for those clinical parameters displaying most deregulation, including (B) aaIPI, and (C) failure-free survival (FFS). Fold changes (FC) are presented in heat maps; red – upregulated, green – down-regulated and black – equal levels.

**Figure S5**. Correlation of response feature analysis to clinical parameters. (A) Number of significant proteins when correlating response feature analysis to clinical parameters, as determined by t-tests. (B) Deregulated proteins when comparing response features for patients with progression of disease during treatment with those without.
