# Supplementary figures and images for: Plasma immunoprofiling of patients with high-risk diffuse large B-cell lymphoma: a Nordic Lymphoma Group study
Source: Blood Cancer J. 2016 Nov 18;6(11):e501–. doi: 10.1038/bcj.2016.113 (PMC5148057; doi:10.1038/bcj.2016.113)

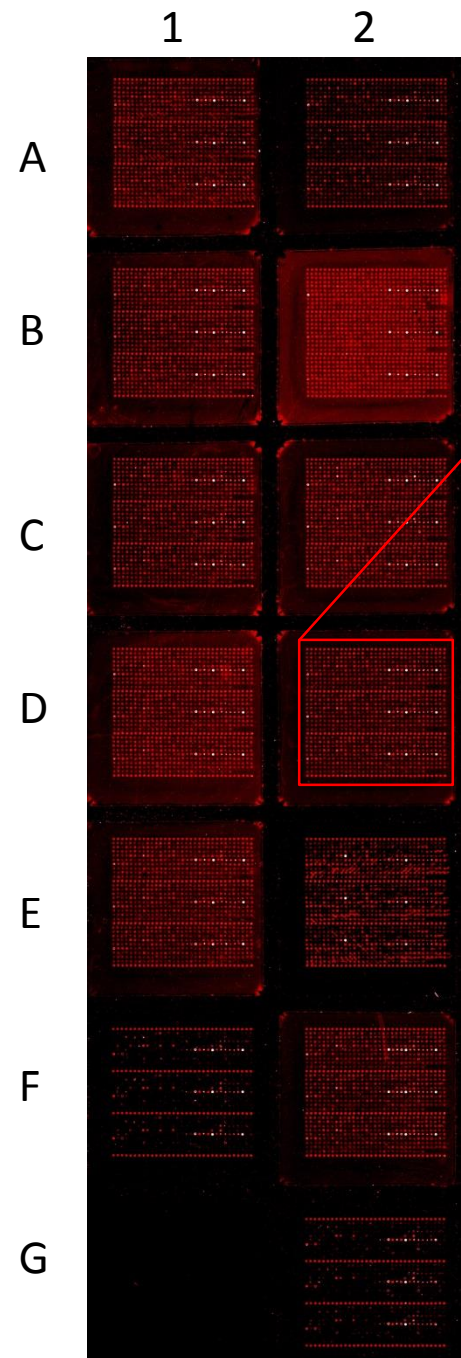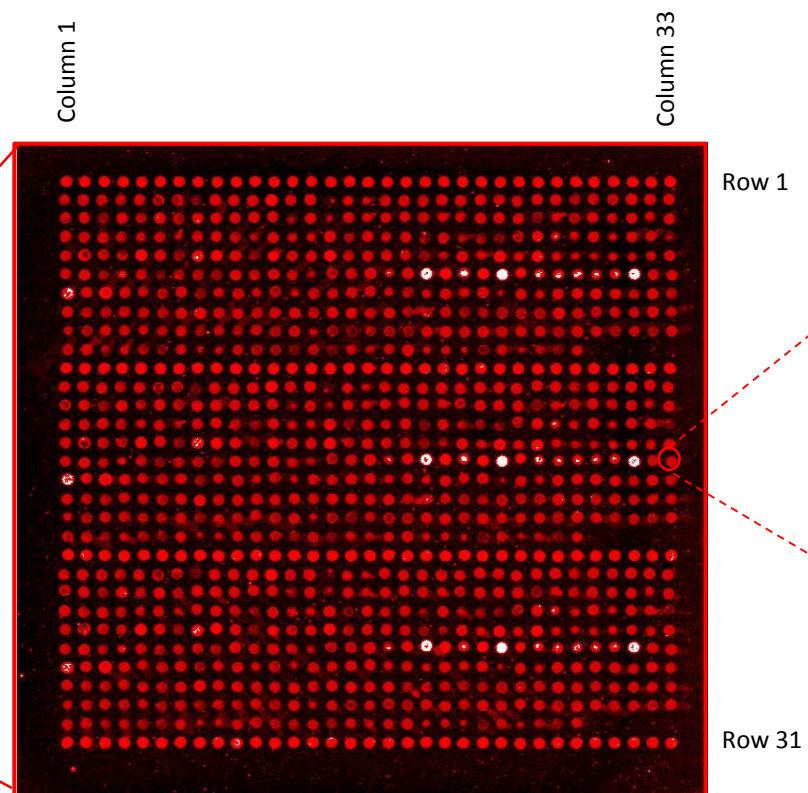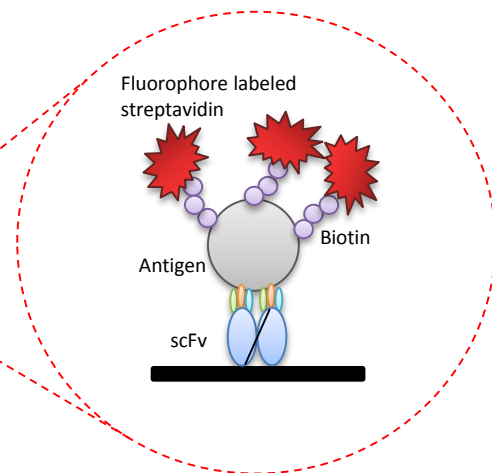

Supplement: Supplementary Figure 1 [file bcj2016113x5.pdf]

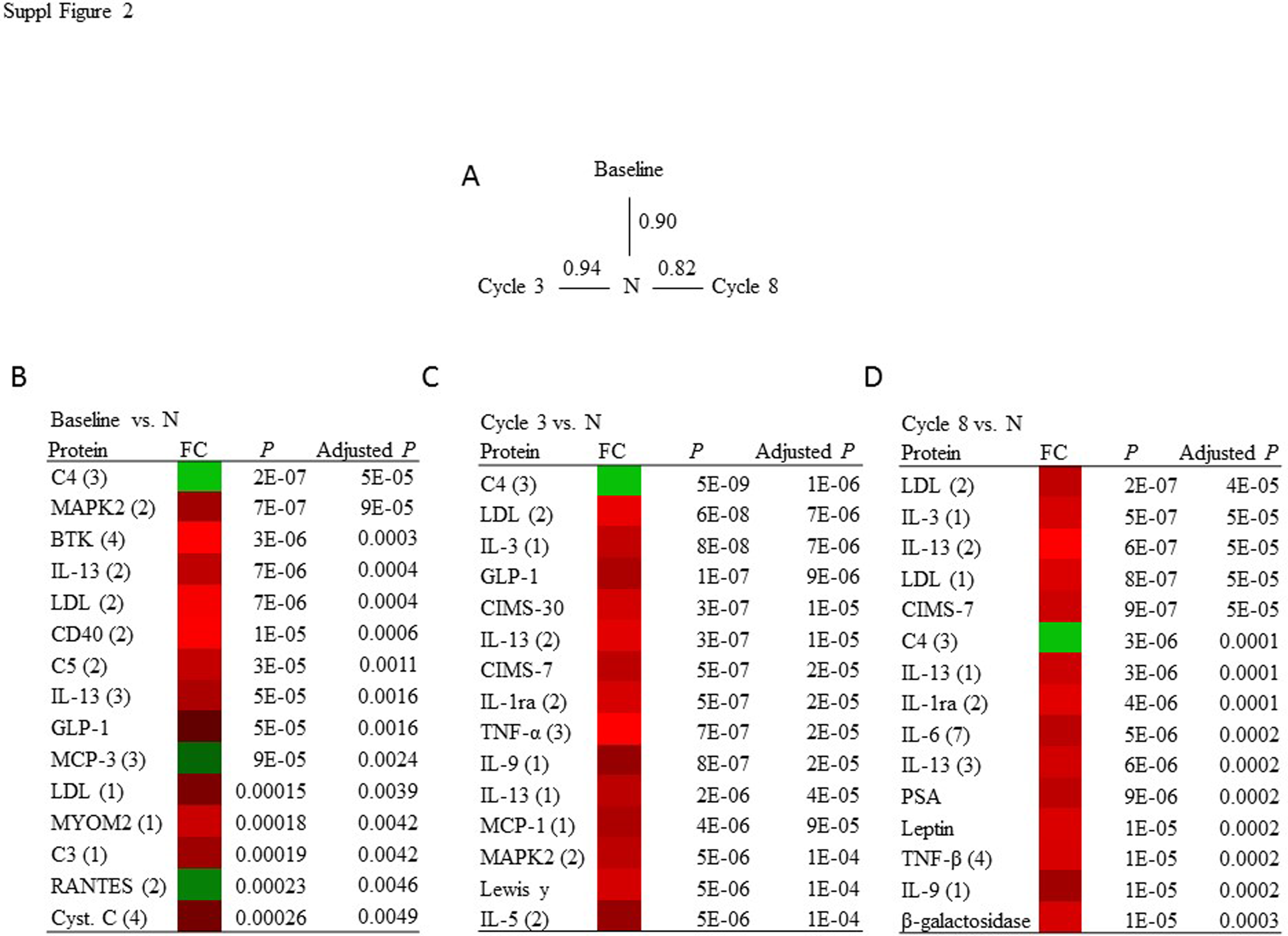

Supplement: Supplementary Figure 2 [file bcj2016113x6.tif]

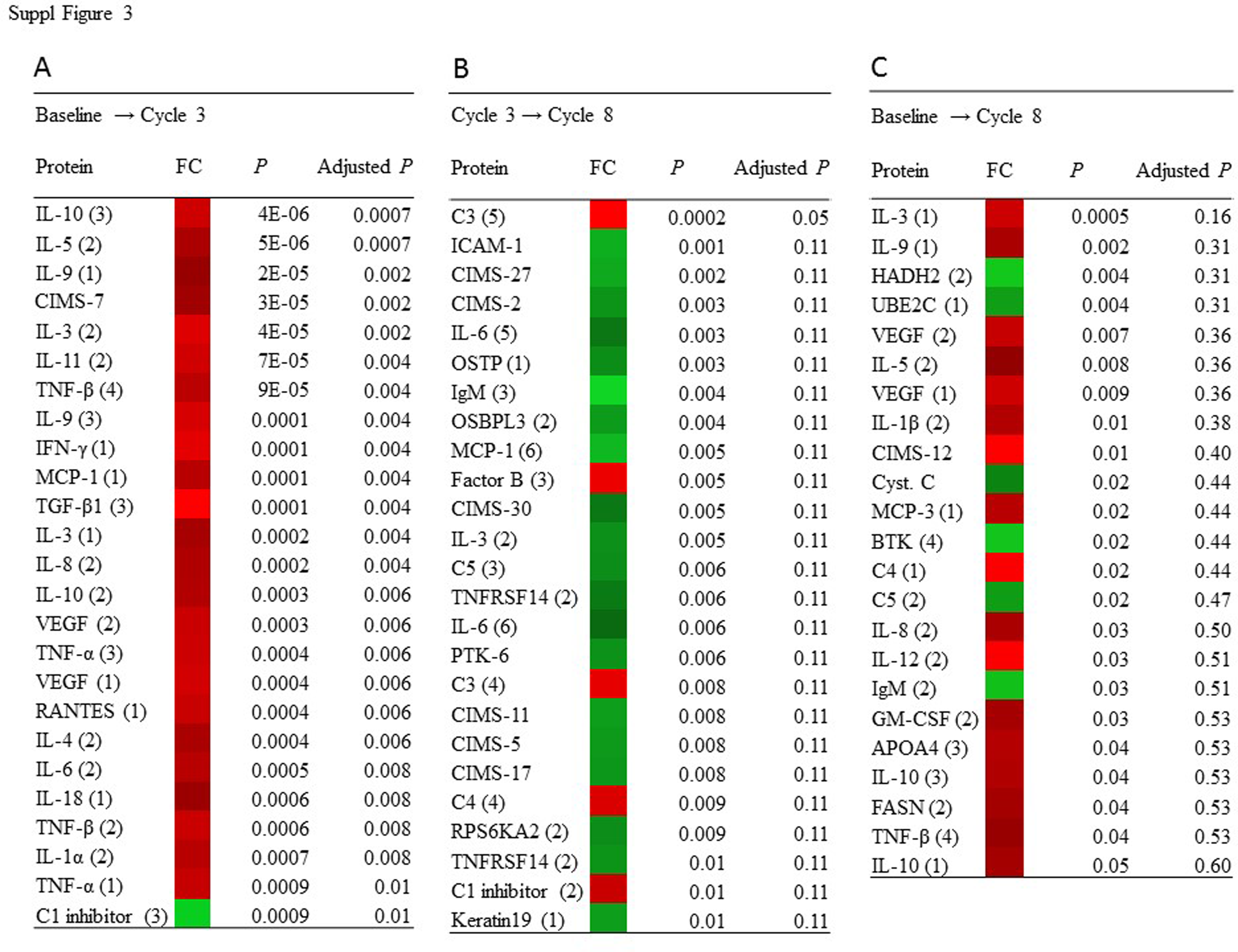

Supplement: Supplementary Figure 3 [file bcj2016113x7.tif]

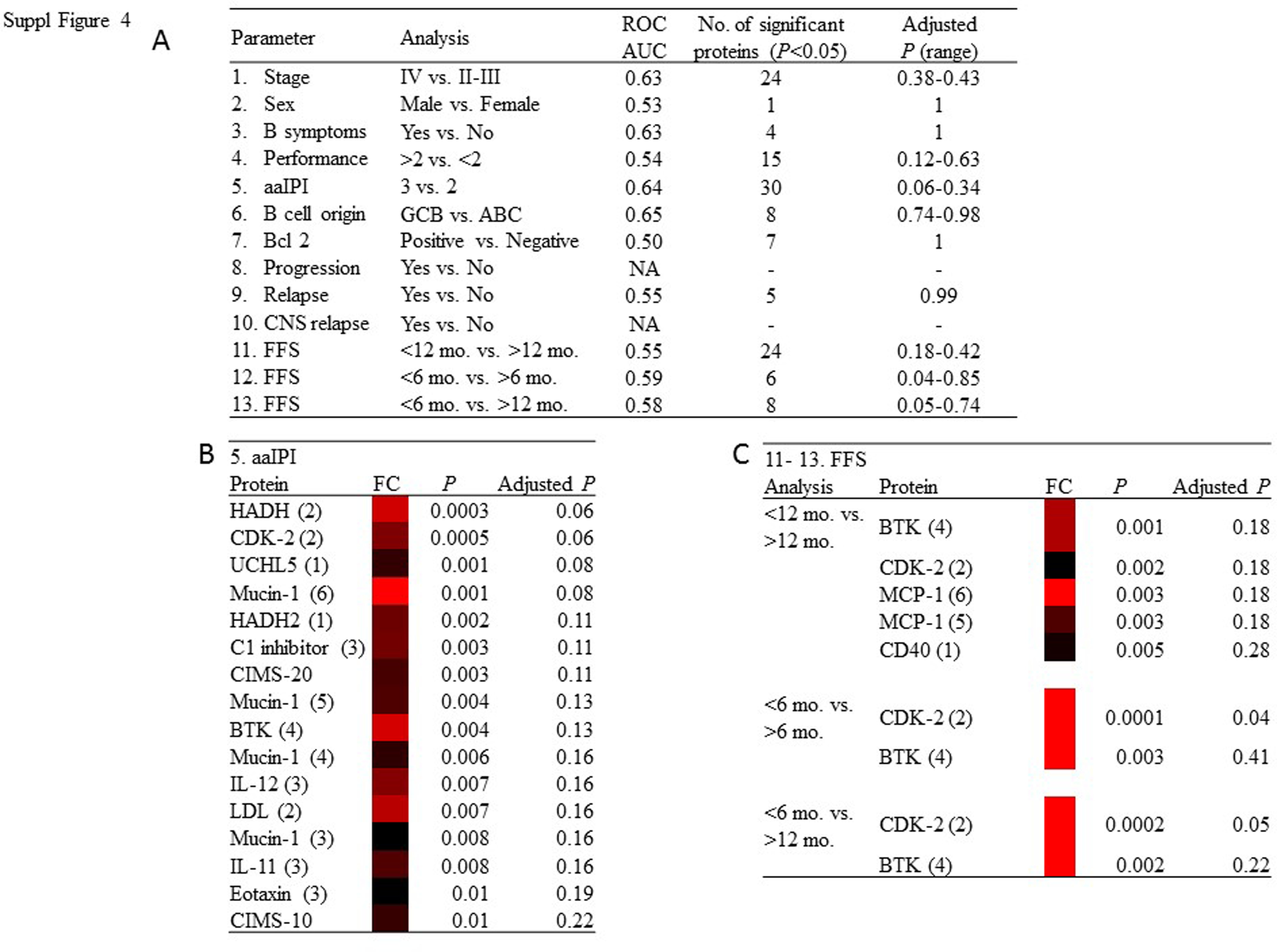

Supplement: Supplementary Figure 4 [file bcj2016113x8.tif]

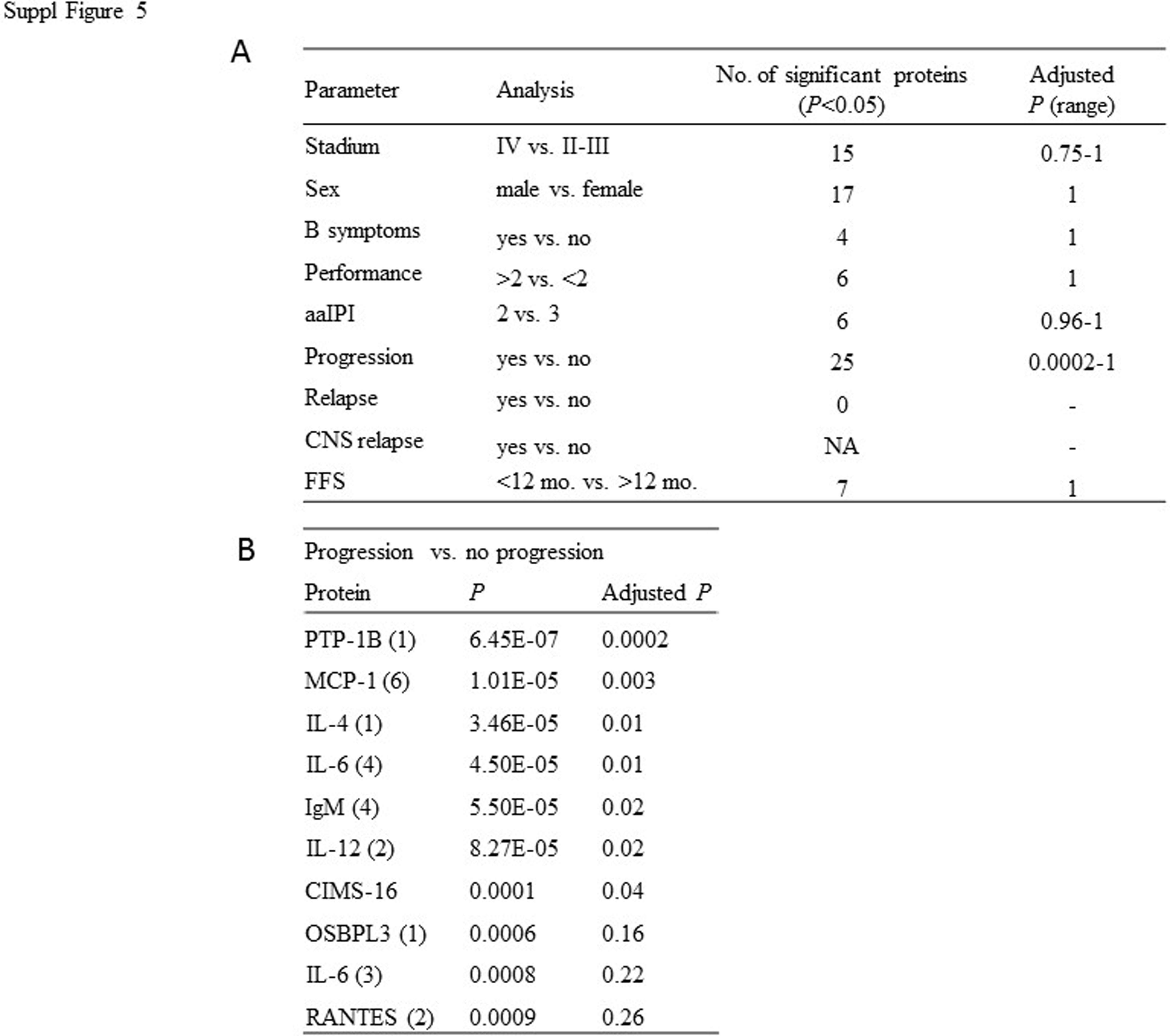

Supplement: Supplementary Figure 5 [file bcj2016113x9.tif]
